# Electrochemical advanced oxidation combined to electro-Fenton for effective treatment of perfluoroalkyl substances “PFAS” in water using a Magnéli phase-based anode

**DOI:** 10.1039/d4na00626g

**Published:** 2024-11-15

**Authors:** Chaimaa Gomri, Elissa Makhoul, Fatou Niang Koundia, Eddy Petit, Stéphane Raffy, Mikhael Bechelany, Mona Semsarilar, Marc Cretin

**Affiliations:** a Institut Européen des Membranes-IEM (UMR 5635), Univ Montpellier, CNRS, ENSCM 34095 Montpellier France mikhael.bechelany@umontpellier.fr; b Gulf University for Science and Technology, GUST Kuwait; c Saint-Gobain C.R.E.E. 550 Avenue Alphonse Jauffret 84300 Cavaillon France

## Abstract

Per-and polyfluoroalkyl substances (PFAS), known as “forever chemicals”, are posing a considerable threat to human health and the environment, that conventional treatment methods are unable to treat. In recent years, electrochemical advanced oxidation emerged as a promising technology for the degradation of recalcitrant pollutants such as PFAS. This work reports the degradation of perfluorooctanoic acid (PFOA) and perfluorooctanesulfonic acid (PFOS), using a Magnéli phase-based anode type Ti_4_O_7_ by electro-oxidation and electro-oxidation combined with electro-Fenton. First the Ti_4_O_7_ anode was prepared from Rutile TiO_2_ powder and characterized, the results showed that the Ti_*n*_O_2*n*−1_ phase is the dominant phase. Afterward, the degradation of PFOA and PFOS was evaluated on the developed anode. After 5 hours of treatment, 52% and 82% of PFOA and PFOS were removed respectively. To improve this results electro-oxidation was combined with electro-Fenton, the degradation of both pollutants increased, 92% of PFOA was degraded and PFOS was totally removed after 5 hours of treatment. The energy consumption was also evaluated at *t*_1/2_ which is defined as the time when half of the initial concentration of PFOA and PFOS was degraded. Combining the two degradation approaches showed promising results that need to be further optimized for potential application at large volumes.

## Abstract 2

This study investigates PFOA and PFOS degradation using a Ti_4_O_7_ anode in electro-oxidation/electro-Fenton processes. The combined treatment achieved 92% PFOA removal and complete PFOS removal in 5 hours, showing potential for large-scale applications.

## Graphical abstract

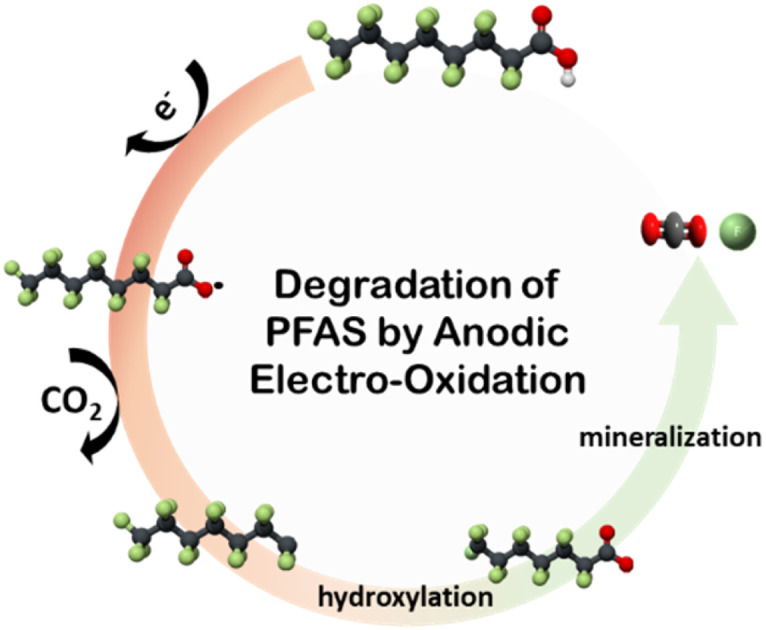

## 1. Introduction

Per- and poly-fluoroalkyl substances (PFAS) are a family of over than 4700 human-made compounds, with unique structures and properties, which favored their use in wide range of products such as photographic materials, cosmetics, firefighting foams, medical devices, *etc.* The extensive use of PFAS induces widespread environmental contamination, increasing of the scientific community's concern regarding their toxicity and persistence in the environment.^1^ PFAS can be fully or partially fluorinated, which gives them good thermal stability and chemical resistance. Furthermore carbon–fluorine bond has the highest bond dissociation energy,^2^ which makes their treatment more challenging compared to other types of pollutants.^3,4^ Perfluorooctanoic acid (PFOA) and perfluorooctanesulfonic acid (PFOS) are two compounds of the PFAS family that are commonly found in the environment.^5,6^ These two compounds have the same tail structure where each hydrogen atom has been replaced by fluorine one, but different head groups. PFOA have a carboxylic head group and PFOS have a sulfonic one.^6,7^

Given their complexes structure, PFAS are difficult to be completely treated with only conventional methods such as adsorption or filtration. Advanced technologies are being explored to ensure the efficient removal and degradation of contaminants. Techniques such as photocatalysis, plasma treatment, and electro-oxidation have been evaluated for their effectiveness in treating pollutants. Photocatalysis offers a sustainable approach that could result in lower costs; however, it operates at slower degradation rates and poses challenges for scaling up.^8^ In contrast, plasma treatments provide rapid and comprehensive solutions but incur higher energy and capital expenses.^9^ Meanwhile, electro-oxidation is emerging as a crucial option due to its cost-efficiency. It is an ideal solution since it does not rely on adding any oxidizing agent.^10^ It involves the oxidation of organic pollutants at the anode of an electrochemical cell, where an electric current is applied to the cell to drive the oxidation reaction.^11^ One main factor that defines the efficiency of this treatment technique is the anode material. There are two types of anodes, “active” and ‘non-active”. Anodes with low oxygen evolution over potential are defined as “active.” Examples are platinum, IrO_2_, RuO_2_, and carbon anodes.^12^ While “non-active” anodes such as boron-doped diamond (BDD) and Magnéli phases of titanium oxide electrode, do not favor the oxygen evolution reaction. Instead, they favor a complete degradation of the pollutants until total mineralization, making them great candidate for water treatment.^13^

Magnéli phases of titanium oxide-based anode has gained a lot of interest due to their low production cost, good chemical stability, high conductivity, and efficiency.^14^ They are a group of compounds composed of a mixture of stoichiometry with the general formula of Ti_*n*_O_2*n*−1_, where *n* can be between 3 and 10. The value of *n* defines the intensity of the electrical conductivity. When *n* = 3–5, the electrical conductivity is the highest, while for values higher than 5, the electrical conductivity decreases.^15^ Ti_4_O_7_ is the Magnéli phase material with the highest electrical conductivity at room temperature.^16^ It has been widely used in electrochemical anodic oxidation to treat a large range of pollutants, like antibiotics,^17^ dyes,^18^ and phenolic compounds,^19^ always exhibiting outstanding results.^20^ Recently there have been reports on efficient degradation of PFAS (up to 90%) using the Magnéli phase based anodes. As it has been reported by Liang *et al.*, PFOA and PFOS degradation can be achieved within 3 hours of electrolysis, which is a relatively short timeframe. Moreover the energy consumption was estimated to be 0.45 kW h per liter which is lower compared to other electrochemical methods, making the process a cost-effective approach for PFAS remediation.^21^

However, this results can still be optimized by combining electro-oxidation to a synergic approach. For instance, Luo *et al.* combined ultrasound irradiation with electro-oxidation to enhance defluorination during degradation through improving the mass transfer and production of radicals.^22^ In another example, Shi *et al.* proved that coupling electrocoagulation to electro-oxidation is useful to overcome the limitation of electro-oxidation to break down PFAS at low concentrations.^23^ One of the most efficient methods used for water remediation is the electro-Fenton process.^24^ The combination of electro-Fenton with other advanced oxidation methods has proven to be highly effective, since the mineralization efficiency is enhanced and the operational costs are reduced.^25^ However, combination of electro-Fenton with electro-oxidation has never been reported for the treatment of PFAS.

This work looks into the degradation of PFOA and PFOS using the combination of electro-oxidation and electro-Fenton to demonstrate the synergic effect of coupling these two technologies. The Ti_4_O_7_ anode was first prepared by plasma deposition. The morphology, composition and the presence of the Magnéli phases of the electrode was confirmed using scanning electron microscopy (SEM), X-ray diffraction (XRD), X-ray photoelectron (XPS) and RAMAN spectroscopies. The degradation of PFOA and PFOS was then evaluated. Factors such as the nature of the PFAS head-group and concentration along with the effect of coupled technology and energy consumption were assessed.

## Material and methods

### Material

Ti_4_O_7_ anode was provided by Saint-Gobain C.R.E.E. Carbon felt was purchased from Alfa Aesar. All chemicals (perfluorooctanoic acid (95%), perfluorooctanesulfonic acid (∼40%), ferrous sulfate (heptahydrate) (FeSO_4_·7H_2_O), anhydrous sodium sulfate (Na_2_SO_4_, 95%) and sulfuric acid) were purchased from Sigma Aldrich. Solutions were prepared using Milli-Q water.

### Anode synthesis

Rutile TiO_2_ powder (from ALTICHEM) was blended with pet coke (from CABOT CORPORATION) and fed into an electrical arcs furnace. The blended powders were melted due to the energy from the electric arcs, and the pet-coke was partially reduced to TiO_2_. The melted composition was poured into a graphite mold. After cooling down, the obtained lingot was jaw-crushed, milled, and sieved to get a powder with particles from 20 to 45 μm. The average sub-stoichiometry of this powder was estimated to be TiO_1.76_, which corresponds to the domain of both Ti_4_O_7_ and Ti_5_O_9_.

The obtained powder was then fed into a plasma torch in which a mixture of Ar and H_2_ was ionized by an electric arc when passing between a tungsten cathode and a copper anode. Due to the extreme temperatures reached in the plasma plume (>10 000 K), the powder was melted. The droplets were accelerated and deposited onto a titanium plate placed in front of the plasma torch. The plasma torch procedure had to be repeated several times to build a coating of about 300 μm thick.

### Physico-chemical characterization of the anode

Surface roughness were obtained using both Bruker's NT1100 and NPFLEX 3D optical profilometers in vertical scanning interferometry. The surface morphology was examined using a Hitachi S4800 scanning electron microscope (SEM) and a three-dimensional (3D) optical microscope (VHX-7000, KEYENCE, Osaka, Japan). Raman spectra were collected using dispersive Raman spectroscopy (HORIBA LABRAM, *λ* = 659 nm) with a fixed laser power of 20 W. The acquisition conditions were, continuous mode of 10 s, a snapshot time of 7 s, and 2.5 accumulations set up to 30 times. To determine the elemental composition of the anode surface, X-ray photoelectron spectroscopy (XPS) was performed using a monochromatic X-ray source (Al-Kα, 1486.6 eV – Resolution FWHM 0.45 eV). X-ray diffraction (XRD) analysis was conducted using a PANAlytical Xpert-PRO diffractometer with an Xcelerator detector, employing Ni-filtered Cu-radiation with a wavelength of 1.54 Å. The scan step size was set to 0.0020889° per step, with a time of 200.660 seconds per step, and the scanning range covered 2*θ* = 20°–80°.

### Electrochemical degradation experiments

#### Anodic electro-oxidation

Electro-oxidation experiments were performed in a cell containing the developed Magnéli phase anode and carbon felt as the cathode. The developed anode had rectangular shape with a total frontal surface of 60 cm^2^ (10 cm × 3 cm × 2) and a thickness of 0.2 cm. Only 30 cm^2^ of this was immersed in the solution. The anode was placed in the middle of the cell. The surface of the Carbon felt was 176 cm^2^ (22 cm × 8 cm), and it was placed all over the cell wall, keeping a distance of 2.5 cm from the anode. The working volume was 200 mL. To ensure ionic transport, Na_2_SO_4_ (50 mM) was added to the solutions of PFOA and PFOS (0.2 ppm and 2 ppm). A current intensity of 0.4 A was supplied by a DC power generator (ELC DC Power supply AL78NX). The experiments were ran for 5 h.

#### 1.4.2. Anodic electro-oxidation combined with electro-Fenton

To combine electro-oxidation with electro-Fenton, FeSO_4_·7H_2_O (0.2 mM) was added, with a drop of sulfuric acid to reach pH = 3. O_2_ (from air liquid) was bubbled during all the experiments to ensure a permanent production of H_2_O_2_.

### 1.5. PFOA and PFOS analysis

In each experiment, samples were taken every 15 minutes for 1 hour and then every hour until the end of the experiment. Concentrations of PFOA and PFOS were evaluated by high-performance liquid chromatography coupled with mass spectroscopy (HPLC-MS). The setup was equipped with Waters-Xselect HSST3 100 mm × 2.1 mm column with 2.5 μm particle size. The mobile phase was composed of Buffer A (water + 0.1% formic acid) and Buffer B (acetonitrile + 0.05% formic acid). The flow rate was 0.25 mL min^−1^.

### Energy consumption

The energy consumed in each treatment method after the degradation of 50% of PFOA and PFOS was calculated using eqn (1):1
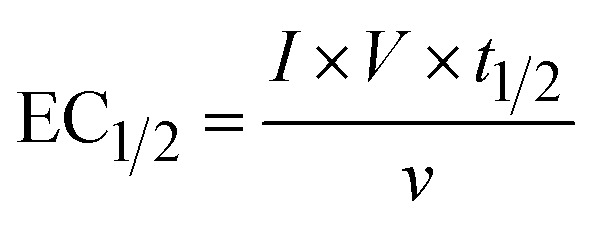
where EC_1/2_ (kW h m^−3^) is the energy consumed to degrade half of the pollutant*, I* (A) is the courant intensity applied, *t*_1/2_ (h) is the time needed to degrade half of the initial concentration of the pollutants, *ν* (L) is the volume treated and *V* (V) is the voltage.

## 2. Results and discussion

### 2.1. Physico-chemical characterization of the anode

**Fig. 1:**
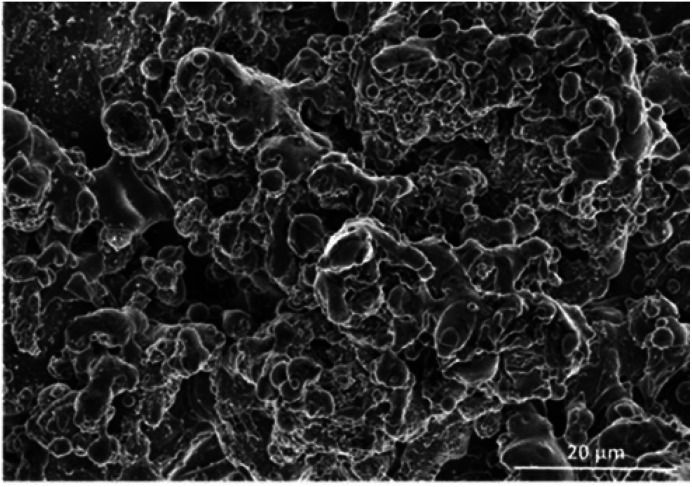
SEM image of the surface of the plasma coated TiO_*x*_.

Fig. 1 is the SEM image of the anode surface, showing TiO_*x*_ particles of around 2–10 microns in size, randomly distributed. This distribution contributes to an increased roughness of the membrane. The roughness of the membrane was approximately 10 673 ± 1230 nm, calculated using profilometer.

**Fig. 2:**
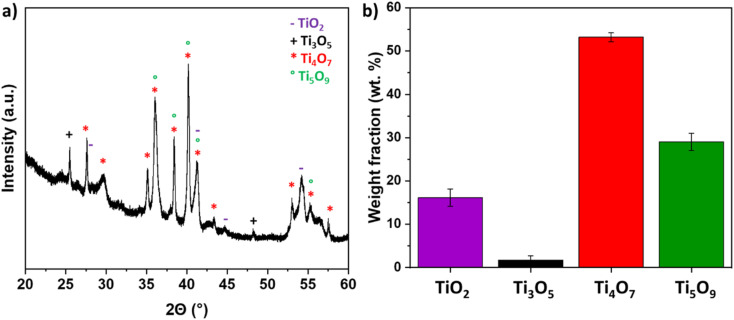
(a) XRD pattern of TiOx sample (b) weight fractions of the formed crystalline phases derived *via* peak deconvolution.

The TiO_*x*_ coating was also analyzed by XRD. The XRD diffractogram showed relatively wide peeks suggesting very small crystallites, most probably due to the extreme quench induced by the contact of the molten particles with the cold metallic substrate. The identified phases were Ti_4_O_7_ (JCPDS card no. 50-0787), Ti_5_O_9_ (JCPDS card no. 51-0641), and Ti_3_O_5_ (JCPDS card no. 1-82-1137), but also TiO_2_ rutile (JCPDS card no. 21-1276) (Fig. 2). The presence of this latter phase can be explained by the turbulences of the plasma plume, leading to introduction of air into the plume and partial re-oxidation of the ceramic material. The fraction of the phase was determined through Gaussian deconvolution. The results confirmed that the Ti_*n*_O_2*n*−1_ phase became the dominant phase. The sample primarily consisted of Ti_4_O_7_ (53.12 ± 1.06 wt%) and Ti_5_O_9_ (29.02 ± 2.36 wt%), with minor impurity phases, including TiO_2_ (16.22 ± 2.61 wt%) and Ti_3_O_5_ (2.902 ± 2.36 wt%).

**Fig. 3:**
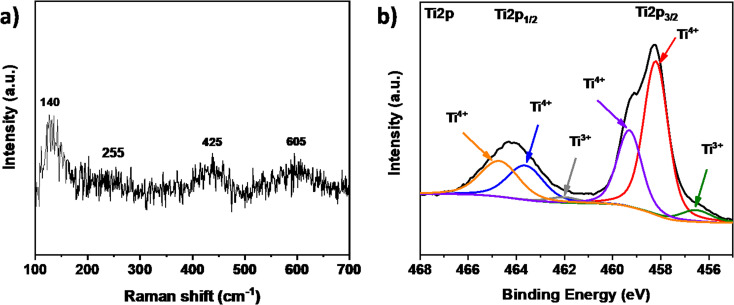
(a) Raman shifts and (b) high-resolution XPS spectra of Ti 2p_3/2_ of the TiOx sample.

Raman spectroscopy of the ceramic material (Fig. 3a) revealed four distinct bands at 140, 255, 425, and 605 cm^−1^. The broad band at 140 is attributed to the metallic high-temperature phase of Ti_4_O_7_, as previously reported.^26^ Additionally, three specific bands at 255, 425, and 605 cm^−1^ were identified as the B_1g_, E_g_, and A_1g_ TiO_2_ rutile modes, respectively.^27^ These results are coherent with the obtained XRD pattern, confirming the presence of the Magnéli as the main phase and the partial re-oxidation of the ceramic material. To confirm the chemical composition and state of the elements, XPS analysis was performed. The results revealed the presence of Ti, and O in different chemical states. Fig. 3b presents the high-resolution Ti 2p spectrum, with the Ti 2p_3/2_ peak at 458.3 eV and the Ti 2p_1/2_ peak at 464.1 eV. Deconvolution revealed six distinct peaks: four at 458.2 eV, 459.3 eV, 464.2 eV, and 465.5 eV, which are attributed to Ti^4+^. This suggests that Ti^4+^ in TiO_2_ exhibits slightly different binding energies compared to Ti^4+^ in Ti_3_O_5_ or Ti_5_O_9_, likely due to defects or neighboring oxidation states. Additionally, two peaks at 456.5 eV and 461.9 eV are indicative of Ti^3+^. These results confirm the presence of different phases characteristic of the Magnéli structure. The dominance of Ti in the 4+ oxidation state over the 3+ state is likely due to the presence of TiO_2_ and the re-oxidation of the Magnéli phase on the surface.

### 2.2. Evaluation of the PFOA and PFOS degradation *via* anodic electro-oxidation

**Fig. 4:**
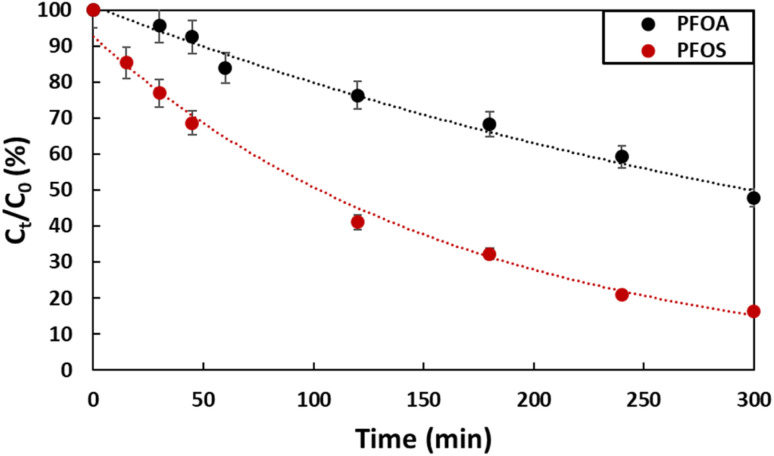
Degradation of PFOA and PFOS (2 ppm) by electro-oxidation using Ti_4_O_7_ as anode with a current density of 13 mA cm^−2^ with Na_2_SO_4_ electrolyte (50 mM).

Degradation of PFOA and PFOS by electro-oxidation was carried out separately, using a solution with initial concentration of 2 ppm for both pollutants. The supporting electrolyte used was Na_2_SO_4_ (50 mM). The experiments were conducted for 5 hours using a current density of 13 mA cm^−2^. This value of current density was selected based on findings from a previous study,^28^ where optimization of the electro-oxidation conditions was carried out to enhance the effective degradation of tetracycline, an antibiotic commonly found as a contaminant in water. The obtained results are presented in Fig. 4 and Table 1. At the end of the experiment, 52% of PFOA was degraded, while in the case of PFOS, the degradation was 82%. The total degradation could have been reached *via* extending the experiment time. PFOS degraded faster than PFOA (*k*_PFOS_ = 0.0064 min^−1^*vs. k*_PFOA_ = 0.0023 min^−1^), despite both being made of 8 carbons. In anodic electro-oxidation, degradation mainly happen at the surface of the anode where hydroxyl radicals are generated according to equation (eqn (2)).

2 Ti_4_O_7_ + H_2_O → Ti_4_O_7_(OH˙) + H^+^ + e^−^

**Table 1:** Comparison of the first-order rate constants of the degradation of PFOA and PFOS (0.2 ppm and 2 ppm) using electro-oxidation for a 5 hour treatment period using a current density of 13 mA cm^−2^

PFAS type	PFOA		PFOS	
Concentration (ppm)	0.2 ppm	2 ppm	0.2 ppm	2 ppm
*k* (min^−1^)	0.0045	0.0023	0.0094	0.0064

Therefore, the efficiency of the degradation depends on the mobility of each compound and its interaction with the anode (Ti_4_O_7_). The degradation pathway using a Ti_4_O_7_ anode involves several steps, as outlined in previous work.^29^ This process entails the gradual cleavage of CF_2_, leading to the breakdown of long-chain PFAS into shorter-chain intermediates that are considerate as less toxic compared to long chain PFAS since they accumulate less in the fatty issues. Initially, perfluorinated radicals are generated through direct electron transfer, which initiates a degradation cycle that continues until complete mineralization is achieved or until short-chain PFAS are produced.^29^ According to Liang *et al.* PFOS gets adsorbed on the surface of the Ti_4_O_7_ much better than PFOA,^30^ since it is a stronger acid (p*K*_aPFOS_ = −3.27, p*K*_aPFOA_ = 0.74–2.58). This strong attachment helps maintaining the negative charge leading to the electro-sorption.^31^

**Fig. 5:**
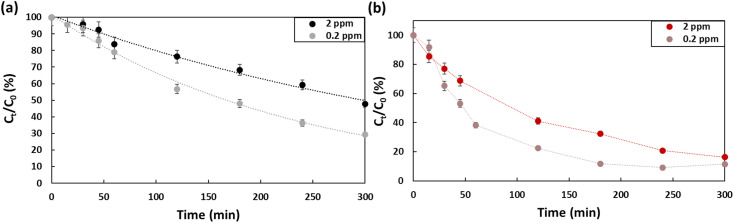
Concentration effect on the degradation efficiency of (a) PFOA, (b) PFOS using Na_2_SO_4_ (50 mM) as electrolyte in a 5 hour treatment period using a current density of 13 mA cm^−2^.

Fig. 5 presents the effect of concentration on the efficiency of the degradation of PFOA and PFOS. Two concentrations were evaluated, 0.2 ppm and 2 ppm. At 0.2 ppm, 60% of the PFOA was degraded (*k*_PFOA-0.2_ = 0.0045 min^−1^), which is approximatively 10% higher compared to the degradation at 2 ppm (*k*_PFOA-2_ = 0.0023 min^−1^). While for PFOS, the difference for the two concentrations is barely distinguishable after 5 hours. Barisci *et al.* suggested that at high initial concentrations, short chain sub-products are produced that would compete with PFOA to interact with hydroxyl radicals, which explain the decrease of PFOA removal.^32^

### 2.3. Evaluation of the degradation of PFOA and PFOS by anodic electro-oxidation coupled to electro-Fenton

**Fig. 6:**
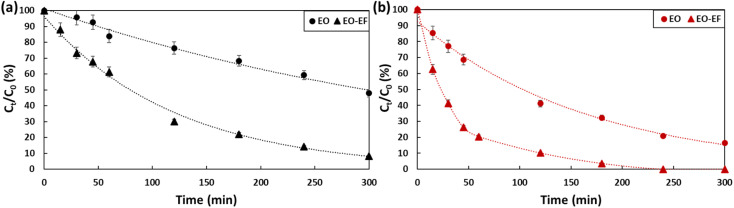
Degradation of (a) PFOA and (b) PFOS (2 ppm) by electro-oxidation and electro-oxidation coupled with electro-Fenton for a 5 hour treatment period using a current density of 13 mA cm^−2^.

To ensure electro-Fenton conditions, FeSO_4_·7H_2_O was added to PFOA and PFOS solutions, pH was adjusted to 3 by adding a drop of sulfuric acid and O_2_ was bubbled during the experiment. Fig. 6 and Table 2 summarize the obtained results for PFOA (a) and PFOS (b). When coupling electro-oxidation with electro-Fenton, 92% of PFOA (*k*_PFOA/EO-EF_ = 0.0084 min^−1^) and 100% PFOS (*k*_PFOS/EO-EF_ = 0.0194 min^−1^) was removed. By coupling the two processes, the degradation of PFOA was enhanced by 40% while PFOS was completely degraded. The combination of electro-oxidation and electro-Fenton favored the generation of hydroxyl radicals on the anode surface as well as on the bulk which is responsible of an efficient degradation. The hydroxyl radicals were generated in the bulk according to eqn (3)–(5). The continuous bubbling of O_2_, generates H_2_O_2_*via* the reduction of O_2_ (eqn (3)). Then, the produced H_2_O_2_ reacts with iron(ii) to generate hydroxyl radicals (eqn (4)). Iron(ii) will be constantly generated due to the reduction of iron(iii) produced (eqn (5)). By synergistically combining electro-oxidation and electro-Fenton techniques, the risk of mass-transfer limitations in mitigated. This combined approach not only promotes the efficient generation of hydroxyl radicals but optimizes their availability in both the bulk solution and at the anode surface, leading to improved degradation rates.

3 O_2_ + 2H^+^ + 2e^−^ → H_2_O

4 Fe^2+^ + H_2_O_2_ + H^+^ → Fe^3+^ + HO˙ + H_2_O

5 Fe^3+^ + e^−^ → Fe^2+^

**Table 2:** First-order rate constants of the degradation of PFOA and PFOS (2 ppm) using electro-oxidation and electro-oxidation combined with electro-Fenton for a 5 hour treatment period using a current density of 13 mA cm^−2^

PFAS type	PFOA		PFOS	
2 ppm	EO	EO-EF	EO	EO-EF
*k* (min^−1^)	0.0023	0.0084	0.0064	0.0194

### 2.4. Energy consumption at 50% degradation of the initial concentration

**Fig. 7:**
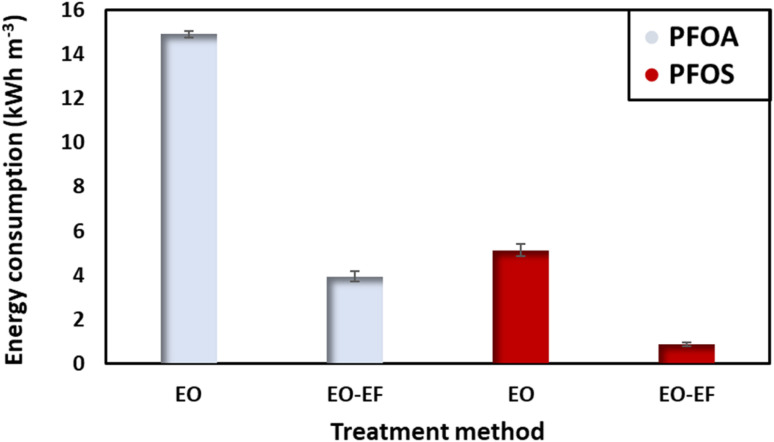
Energy consumed at *t*_1/2_, after the degradation of 50% of the initial concentration (2 ppm) using a current density of 13 mA cm^−2^.

Energy consumption is a crucial parameter to evaluate the feasibility of the process in terms of environmental impact and the operational costs. In this context, the energy consumed to degrade PFOA and PFOS at *t*_1/2_, corresponding to the degradation of half of the initial concentration was evaluated (Fig. 7). By comparing the energy consumed to degrade PFOA and PFOS, it can be stated that PFOS requires less energy for degradation. Also, PFOS tends to degrade faster than PFOA. For an initial concentration of 2 ppm using only electro-oxidation, PFOA consumes 14.91 kW h m^−3^. While when combining electro-oxidation with electro-Fenton, the energy consumption was 3.7 times lower (3.95 kW h m^−3^). In the case of PFOS, the energy consumed by electro-oxidation was 5.14 kW h m^−3^ (3-times less as compared to the energy needed to degrade PFOA). Combining electro-oxidation with electro-Fenton enhances the degradation efficiency of PFAS and reduces the energy consumption which is an important parameter to consider for large scale water treatment. Coupling the two approaches also reduced the time needed for the degradation. A factor with direct impact on the energy consumption. It is difficult to compare the obtained results with reports in the literature, given the number of different parameters influencing the energy consumption. Table 3 displays the energy consumed to degrade PFOA and PFOS by electro-oxidation using different anodes, providing an approximate reference point in comparison to other studies.

**Table 3:** Comparison of the energy consumption for PFOA and PFOS degradation using different types of anodes

Pollutant	Concentration	Removal rate (%)	Energy consumption	Anode	Current density	Ref.
PFOA	0.5 mM	99.9	14.2–76.2 W h L^−1^	Ti_4_O_7_	5 mA cm^−2^	33
PFOS	0.1 mM	93.1	36.9–820 W h L^−1^			
PFOA	1350 ng L^−1^	80	88–114 kW h m^−3^	BDD	75 mA cm^−2^	34
PFOS	3280 ng L^−1^	78	123–108 kW h m^−3^			
PFOA	1 μg L^−1^	72	164.9 kW h m^−3^	Ag/Au-PAA/PAH	10 mA cm^−2^	35
PFOS	1 μg L^−1^	91	90 kW h m^−3^			
PFOA	2 ppm	50	14.91–3.95 kW h m^−3^	Ti_4_O_7_	13 mA cm^−2^	This study
PFOS	2 ppm	50	5.14–0.89 kW h m^−3^			

## 3. Conclusion

This work reports the preparation of Magnéli phase based anode and its application in electro-oxidation to degrade PFOA and PFOS. The anode was prepared by oxidation of rutile using plasma torch, the XRD analysis showed the presence of different Magnéli phase with Ti_4_O_7_ being dominant. Then, the performance of the anode has been evaluated by electro-oxidation, the anode demonstrated great stability, performing consistently over several electro-oxidation cycles, which indicates that its structural integrity, electrochemical behavior, and chemical properties were preserved. The anode can continuously operate without the need for frequent replacements or repairs. Moreover, no fouling has been observed, implying that the active sites were not blocked. This highlights the cost-effectiveness of using a Magnéli phase-based anode. The degradation rate of PFOA at an initial concentration of 2 ppm was 60% using electro-oxidation. However, when electro-oxidation was combined to electro-Fenton, the degradation rate increased 92%. A similar effect was observed for PFOS, where the combination of these two approaches led to complete degradation of PFOS. Although, PFOS was degraded more readily than PFOA; this difference in degradation kinetic was reported to be due to the interaction of each compound with the anode. In fact, PFOS is more acidic, so it easily gets adsorbed on the anode. The concentration of the compounds, impact the degradation efficiency. At higher concentrations, and for anodic oxidation the degradation was less efficient than at lower concentrations because of the competition effect of the generated by-products. In summary, the degradation of PFOA and PFOS was more efficient when electro-oxidation was coupled with electro-Fenton. This synergic approach led to enhanced removal of these recalcitrant pollutant. Hydroxyl radicals were generated not only on the surface of the anode but also in the bulk, which reduces the mass-transfer limitation and change the dependence with the concentration of the pollutant. Furthermore, coupling the two techniques increases the cost-efficiency because of higher degradation kinetic, so energy consumption was up to 3 times lower than electro-oxidation approach. Additional research is needed to improve electrode design and explore alternative strategies, such as the usage of tubular anode or incorporating multiple anodes, to create more sustainable electrochemical process. Adjusting operating parameters such as pH, current density and temperature of electro-oxidation can enhance reaction kinetics by providing additional energy to overcome activation barriers.

## Data availability

The datasets generated and/or analyzed during the current study are available from the corresponding author on reasonable request.

## Conflicts of interest

The authors declare no conflict of interest.
